# Compositional Studies: Antioxidant and Antidiabetic Activities of *Capparis decidua* (Forsk.) Edgew

**DOI:** 10.3390/ijms12128846

**Published:** 2011-12-05

**Authors:** Muhammad Zia-Ul-Haq, Sanja Ćavar, Mughal Qayum, Imran Imran, Vincenzo de Feo

**Affiliations:** 1 Department of Pharmacognosy, Research Institute of Pharmaceutical Sciences, University of Karachi, Karachi-75270, Pakistan; E-Mail: ahirzia@gmail.com; 2 Department of Chemistry, University of Sarajevo, Sarajevo-71000, Bosnia and Herzegovina; E-Mail: sanja.cavar@pmf.unsa.ba; 3 Department of Pharmacy, Anbar Campus, Abdul Wali Khan University, Mardan 23200, Pakistan; E-Mail: mu_afridii@yahoo.com; 4 Faculty of Pharmacy, Bahauddin Zakariya University, Multan 60800, Pakistan; E-Mail: imran.ch@bzu.edu.pk; 5 Department of Pharmaceutical and Biomedical Sciences, University of Salerno, Salerno 84122, Italy

**Keywords:** *Capparis decidua*, nutritional constituents, amino acids, sterols, tocopherols, phenols, antioxidant activity, antidiabetic activity

## Abstract

*Capparis decidua* is one of the traditional remedies used for various medicinal treatments in Pakistan. This study presents the determination of proximate composition, amino acids, fatty acids, tocopherols, sterols, glucosinolate and phenolic content in extracts obtained from different aerial parts of *C. decidua*, as well as their antidiabetic and antioxidant activity. All examined extracts were prominently rich in phenolics and glucosinates, and they showed potent antidiabetic and antihemolytic activity. The present study could be helpful in developing medicinal preparations for the treatment of diabetes and related symptoms.

## 1. Introduction

*Capparis decidua* (Forsk.) Edgew (Capparidaceae), locally known as Kair, is a drought resistant plant growing in dry regions of Pakistan as dense tufts [1]. Besides many socioeconomic and ecological benefits [2], all parts of this plant have a number of medicinal properties. The plant is traditionally used to cure toothache, arthritis, asthma, cough, inflammation, intermittent fevers, malaria, rheumatism, and swelling. It is also believed to possess laxative, astringent and vermifuge properties [3,4]. The alcoholic extract of fruit pulp and root bark is claimed to have anthelmintic activity. The fruits and the seeds are used to cure cholera, dysentery and urinary purulent discharges and have diuretic and antidiabetic properties [5]. The spicy taste fruits serve as an astringent for bowels, a remedy for bad breath and is claimed to cure cardiac troubles [4]. The green immature fruits are considered antihelminthic and laxative and are employed in the treatment of asthma, constipation, coughs, hysteria and other psychological problems [6]. The blanched fruit is used as a vegetable [7]. Green berries are used in food preparations such as pickles [8]. The seeds oil is edible when processed and also used to cure skin diseases [4].

Several chemical and pharmacological researches have been carried out on *C. decidua* Sterols [9], fatty acids [10], flavones [11], oxygenated heterocyclic constituents [12], alkaloids [13–18], and an isothiocyanate glucoside [19] have been reported in different parts of this plant. The nutritional value of flowers and fruits of *C. decidua* was also evaluated [20,21].

Different extracts of the plant have been demonstrated to possess pharmacological properties. The plant has been reported for its Central Nervous System sedative and depressant [22,23], and antimicrobial properties [8,24,25]. Methanol and water extracts of *C. decidua* possessed hepatoprotective activity [26]. The effects of extracts of the plant on human plasma triglycerides, total lipids and phospholipids have been reported [27]. The fruit has been shown to possess anti-atherosclerotic [28], antidiabetic [29,30], anti-hypertensive [31], and anti-hyperlipidemic [28,32,33] properties. The cardiovascular activity of capparidisine, a spermidine alkaloid from *Capparis decidua*, has been reported [34].

This study reports some chemical features of this plant, along with the evaluation of antioxidant, antidiabetic and antihemolytic activities of extracts of different parts of *C. decidua.*

## 2. Results and Discussion

To the best of our knowledge, there is no previous report on compositional studies of *C. decidua* seeds. These were firstly subjected to proximate analysis. Results indicate presence of high amounts of carbohydrates (25.42 ± 0.26%), proteins (27.71 ± 1.39%), and lipids (29.11 ± 1.07%) (Table 1).

Proximate composition is therefore an index of total energy content in a food and its analysis usually is the first step when evaluating its nutritional potential. Our results agree with those reported earlier for other parts of *Capparis decidua* and for other *Capparis* species [35,36]. A balanced amino acid profile is an indicator of quality of proteins and foods. The amino acid content of *C. decidua* seeds (Table 2) indicated that glutamic (24.01 ± 0.56%) and aspartic acids (11.91 ± 0.14%) were present in highest concentrations, while methionine (0.75 ± 0.62%) and cysteine (0.34 ± 0.01%) were in lowest concentrations. A similar amino acid pattern was reported for other *Capparis* species [37].

Fatty acid composition (Table 3) showed a high content of linoleic acid (47.33 ± 1.04%), while eicosenoic acid has been found in lowest amounts (0.52 ± 0.38%). These results are similar to the data previously reported [38,39]. It has been reported that linoleic acid prevents cardiovascular disorders such as coronary heart disease, atherosclerosis, as well as hypertension [40]. Fatty acid composition of *Capparis decidua* seed oils can be considered an interesting point with regard to the further use of the seeds for oil purpose.

Moreover, γ-Tocopheol was found in highest amount in seed oil (Table 4), while β-tocopheol was found in lowest amount. These results are similar to those reported for *Capparis spinosa* [41]. High amounts of tocopherols can be interesting for the stabilization of fats and oils against oxidative deterioration and for applications in dietary, pharmaceutical, or biomedical products. Sterol profile of *Capparis decidua* seed oil indicated that β-sitosterol was the major constituent (Table 5). Like other parameters, no previous study reported sterol contents of this species. Sterols are perhaps the most important class of the minor components and comprise a major portion of the unsaponifiable matter of most vegetable oils. The occurrence of Δ^5^-avenasterol in the seed oil is of interest because this compound is known to act as an antioxidant and as an anti-polymerization agent in frying oils [42]. Sterols with an ethyldiene group in the side chain are believed to be one of most effective antioxidants.

Plants producing large amounts of glucosinolates are of interest, because their derivatives can serve as natural pesticides and are under investigation as antitumoral drugs [43]. Glucosinolate contents indicated that all parts of plant material of *C. decidua* contained relatively high amounts of these anti-nutrients (Table 6). Glucosinolates are present in highest concentration in stems (107.39 ± 1.57 μmol/g) while lowest amount was detected in roots (77.48 ± 0.59 μmol/g). Glucosinolates enzyme degradation products produced negative sensory properties in *Capparis decidua* and may have harmful effects on animal health. Microorganisms invading animal gut enable glucosinolates decomposition and formation of various glucosinolates hydrolysis products, namely isothiocyanates, nitriles and thiocyanates [44]. These compounds, depending on their type, have strong goitrogenic effects and have been frequently reported to be implicated in anti-thyroidal activity [45]. Although glucosinolates have been detected in other *Capparis* species [41,46,47], to the best of our knowledge, there is no report on glucosinolate contents of *Capparis decidua*.

The content of total phenolic compounds of different aerial parts of *C. decidua* is shown in Table 6. Generally, examined extracts showed prominently high levels of phenolic compounds. The highest value was obtained in fruit extract (407.72 ± 0.81 mg CE/g), and the lowest in leaves extract (286.51 ± 4.62 mg CE/g). Our results are in agreement with those found for other *Capparis* species [48,49]. The Folin-Ciocalteu method is relatively simple; however, it is not specific. Heterogeneity of phenolic compounds and the presence of easily oxidized substances other than phenols like vitamin C and Cu(I), leading to elevated phenolic concentrations [50]. Moreover, this method relies on reaction kinetics and not stoichiometric conversion, it is not very precise, and variations of approximately 5% are typical for replicates, depending on the temperature control and timing precision of the reagent additions and spectral measurements [51]. Despite the undefined chemical nature of Folin-Ciocalteu Reagent (FCR), the total phenols assay by FCR is convenient, simple, and reproducible and it has become a routine assay in studying phenolic antioxidants [52].

Three different aerial parts (leaves, flowers and fruits) were subjected to antioxidant activity screening, using different testing methods. The extracts of *C. decidua* showed potent antioxidant activity, reducing different types of radicals (Table 7). In fact, to varying extents, the tested extracts were able to reduce the stable 1,1-diphenyl-2-pictylhydrazyl (DPPH) radical, reaching IC_50_ values from 69.1 ± 1.3 μg/mL, for fruit extract, to 104.17 ± 1.68 μg/mL, for leaves extract. Although DPPH and ABTS (2,2′-azinobis(3-ethylbenzo-thiazoline-6-sulphonic acid) diammonium salt) methods are based on the same principle, data obtained from ABTS assay were lower than those obtained from DPPH assay, reaching Trolox equivalent (TE) values from 341.86 ± 1.37 μmol TE/g for leaves extract, to 501.17 ± 1.34 μmol TE/g for fruit extract. This is probably due to steric factors that are one of the major factors influencing the reduction of stable DPPH radical. Moreover, results obtained from Ferric reducing antioxidant power (FRAP) and Total radical-trapping antioxidant parameter (TRAP) assays are in agreement with values obtained from the above discussed assays. The superoxide radical scavenging assay (SRSA) is said to be more relevant than those methods described above, because it utilizes a biologically relevant radical source. This radical mediates inflammatory tissue injuries in ischemia-reperfusion, arthritis, gout and gastric ulceration. Superoxide radical has a low reactivity and a low capacity to penetrate the lipidic membrane layer, but it can generate hydrogen peroxide and highly reactive hydroxyl radical, via Haber-Weiss reaction. Our findings are in the agreement with those published earlier [29,53].

Erythrocyte membrane, being rich in polyunsaturated fatty acids, is susceptible to free radical mediated peroxidation. Since this peroxidation is a free radical chain reaction, the erythrocyte membrane is quickly damaged leading to hemolysis. Moreover, the red blood cells hemolysis is claimed to be a more sensitive system for evaluating the antioxidant properties of the phytoceuticals [54]. In the present study, the investigated extracts were reported to possess dose dependent inhibitory effects towards hemolysis of cow blood erythrocyte. It is possible that a high total phenol content in the extract can produce this potent antihemolytic activity, as previous studies ascribe the antihemolytic capacity to these components [55].

Diabetes mellitus is a metabolic disorder characterized by a congenital (type I insulin-dependent diabetes mellitus) or acquired (type II noninsulin-dependent diabetes mellitus) inability to transport glucose from the bloodstream into cells [56]. Currently, much attention is being paid to plants and their constituents for the treatment of diabetes. A possible therapeutic approach for treating diabetes is to decrease postprandial hyperglycaemia. This can be achieved by delaying the absorption of glucose through the inhibition of carbohydrate hydrolyzing enzymes, α-amylase and α-glucosidase in the digestive tract [57]. Therefore, α-amylase and α-glucosidase inhibitors found in medicinal plants have long been studied to evaluate their antidiabetic properties. Among the extracts of *C. decidua* studied, the fruit extract showed satisfactory inhibitory effect on both enzymes, followed by flowers and leaves extracts, at both concentrations tested (Table 8). Like other parameters, enzyme inhibition activities of this plant have not been previously reported. Results obtained in the present study support the use of this plant as a dietary supplement for the treatment of diabetes. Further studies are required to find out the mode of action of these plant extracts in inhibiting α-amylase and α-glucosidase.

## 3. Experimental Section

### 3.1. Plant Material

Plant material of *Capparis decidua* (flowers, fruits, stems, and leaves) was obtained from Department of Agronomy, Bahauddin Zakariya University, Multan, Pakistan. Seeds were stored in stainless-steel containers at 4 °C prior to analysis.

### 3.2. Chemicals

6-Hydroxy-2,5,7,8-tetramethylchroman-2-carboxylic acid (Trolox^®^), 2,2′-azinobis(3-ethylbenzo-thiazoline- 6-sulphonic acid) diammonium salt (ABTS) and 2,4,6-tripyridyl-*s*-triazine (TPTZ) were purchased from Sigma-Aldrich (St. Louis, MO). R-Phycoerythrin (R-PE) was purchased from Prozyme (San Leandro, CA); 2,2′-azobis (2-amidinopropane) dihydrochloride (ABAP) was purchased from Waco Chemicals (Richmond, VA). High-purity water was produced in the laboratory by using an Alpha-Q system (Millipore, Marlborough, MA).

### 3.3. Proximate Analysis

Moisture, lipids, ash, protein, and carbohydrates in seeds were determined according to AOAC methods [58].

### 3.4. Amino Acid Analysis

Seed samples (300 g), were hydrolyzed with 6 M HCl in a test tube for 24 h at 105 °C. The dried residue was dissolved in citrate buffer (pH 2.2) after flash evaporation. Aliquots were analyzed in an automatic amino acid analyzer (Hitachi Perkin-Elmer Model KLA 3B), using a buffer system as described earlier [59,60]. Methionine and cystine were separately analyzed after treatment with performic acid and subsequent hydrolysis with HCl. Tryptophan was determined after alkali (NaOH) hydrolysis by the colorimetric method [61].

### 3.5. Fatty Acid Composition of Seeds

The oil was extracted from the ground seeds by extraction with petroleum ether in a Soxhlet apparatus for 6 hr following the AOCS official method [62] The obtained oils were stored at 4 °C until further investigation. The fatty acid composition of oils was determined following the ISO draft standard ISO/FIDS 5509 [63]. In brief, one drop of the oil was dissolved in1 mL of *n*-heptane, 50 μL 2 M sodium methanolate in methanol were added, and the closed tube was agitated vigorously for 1 min. After addition of 100 μL of water, the tube was centrifuged at 4500 g for 10 min and the lower aqueous phase was removed. After that, 50 μL 1 M HCl were added to the heptane phase, the two phases were shortly mixed and the lower aqueous phase was rejected. About 20 mg of sodium hydrogen sulphate (monohydrate, extra pure, Merck, Darmstadt, Germany) were added, and after centrifugation at 4500 g for 10 min, the top *n*-heptane phase was transferred into a vial and injected in a Varian 5890 gas chromatograph equipped with a capillary column, CP-Sil88 (100 m, 0.25 mm ID, film thickness 0.2 μm). The temperature programme was as follows: from 155 °C heated to 220 °C (1.5 °C/min.), 10 min isotherm; injector 250 °C, detector 250 °C; carrier gas 1.07 mL/min hydrogen; split ratio 1:50; detector gas 30 mL/min hydrogen; 300 mL/min air and 30 mL/min nitrogen; manual injection volume less than 1 μL. The integration software computed the peak areas and percentages of fatty acid methyl esters (FAME) were obtained as weight percent by direct internal normalization.

### 3.6. Tocopherol Content in the Seed Oil

For determination of tocopherols, a solution of 250 mg of studied samples oil in 25 mL *n*-heptane was directly performed by HPLC. This analysis was conducted using a Merck-Hitachi low-pressure gradient system, fitted with an L-6000 pump, a Merck-Hitachi F-1000 Fluorescence Spectrophotometer (detector wavelengths for excitation 295 nm, for emission 330 nm) and a D-2500 integration system. 20 μL of the samples were injected by a Merck 655-A40 Autosampler onto a Diol phase HPLC column (25 cm × 4.6 mm i.d., Merck, Darmstadt, Germany) using a flow rate of 1.3 mL/min. The mobile phase used was *n*-heptane/*tert*-butyl methyl ether (99 + 1, v/v) [64].

### 3.7. Sterol Composition in Seed Oil

The determination of sterols was made following the official method of the Association of Official Analytical Chemists [58]. Analysis was carried out on a Perkin Elmer gas chromatograph model 8700, equipped with a methylphenyl polysiloxane coated capillary column OV-17 (30 m × 0.25 mm i.d., 0.20 μm film thickness) and a Flame-Ionization Detector (FID). The column was isothermally operated at 255 °C. Injector and FID temperatures were set at 275 and 290 °C, respectively. Extra pure N_2_ at a flow rate of 3 mL/min was used as a carrier gas. The internal standard used was 5-α-cholestane. Identification and quantification of sterols were performed using a sterol standard mixture.

### 3.8. Sample Preparations and Determination of Glucosinolates

#### 3.8.1. Sample Preparation for Glucosinolates Assay

To prevent enzymatic degradation by myrosinase, all parts of plant material were firstly heated for 2 h at 100 °C, and homogenised directly in an analytical grinder. Grounded material was passed through a 60-mesh sieve. From resulting powder material, 0.100 g was extracted three times (for 15 min each) with 45 mL deionised water at 50 °C. Each mixture was sonicated and centrifuged for 15 min at 5000 rpm. Extract was collected into volumetric flask and the volume was adjusted with deionised water to 50 mL.

#### 3.8.2. Determination of Glucosinolates

The total content of glucosinolates was determined by the slightly modified method proposed by Hu and co-workers [47], using sinigrin as an external standard. The reaction system contained, in a final volume of 8.0 and 2.0 mL of standard or extract, 4.0 mL of 0.1% (w/w) sodium carboxy methyl cellulose (SCMC) in deionised water and 2.0 mL of 0.004 N palladium chloride in 2 N HCl. The reactions were carried out at 25 °C for 1 h in test tubes. The linear range and the equations of linear regression were obtained through such a sequence of 0.05, 0.10, 0.15, 0.20 and 0.25 mg/mL. Mean absorbance (*n* = 3) generated from the standard solutions were plotted against concentration to establish calibration equations. The mixture was decanted into 1 cm light-path cuvettes, and the absorbances at 520 nm were determined against a blank. Each set of determinations also included a control vessel that contained the palladium chloride reagent but no glucosinolates [65]. The equation generated by linear regression was used to establish the concentrations of glucosinates in samples.

### 3.9. Sample Preparation for Determination of Total Phenolic Content and Biological Activity

Different parts of *C. decidua* (leaves, flowers, fruits) were extracted with methanol and water at room temperature. The methanol extracts were filtered and evaporated under vacuum to obtain a thick gummy mass which was tested for total phenolic content, and for antidiabetic and antioxidant activities.

#### 3.9.1. Determination of Total Phenolic Content

A weighed amount of extract sample (between 50 and 130 mg depending on the sample) was dissolved with 10 mL of acidified methanol (1% formic acid). The extracts were kept at 4 °C at dark prior to the analysis. The content of phenolic compounds was determined using the Folin-Ciocalteu method, based on the reduction of phosphowolframate-phosphomolybdate complex by phenolics to a blue reaction product [66]. A 1.0 mL of sample solution was diluted with 60 mL of distilled water, and the 5 mL of Folin-Ciocalteu reagent, previously diluted 2 times, and mixed. After 5–10 min a 15 mL of 20% solution of sodium carbonate was added, and obtained solution was diluted to 100 mL. Prepared samples were kept for 2 h at room temperature and the absorbance was measured at 760 nm. The data were calculated according to standard curve of catechin (0.01–0.20 mg/mL) and they were expressed as catechin equivalents (CE) per gram of dry extract.

#### 3.9.2. Antioxidant Activity

Plant extracts were analyzed for their antioxidant capacity by five different assays: 1,1-diphenyl-2-picrylhydrazyl radical-scavenging activity (DPPH method) [67]; 2,2′-azino-bis(3-ethylbenzthiazoline-6-sulphonic acid) radical-scavenging activity (ABTS method) [68]; ferric reducing antioxidant power (FRAP) assay [69]; total radical-trapping antioxidant parameter (TRAP) assay [70]; superoxide radical scavenging assay (SRSA) [71]. The ABTS and TRAP values were expressed as micromoles of Trolox per gram of plant extract, FRAP values were expressed as micromoles of Fe^2+^ equivalents per gram of plant extract and the DPPH and superoxide radical scavenging activities were expressed as the concentration of sample necessary to decrease by 50% the initial absorbance of radical (IC_50_).

#### 3.9.3. Antihemolytic Activity

The antihemolytic activity of the plant extracts of *C. decidua* was performed as described by Naim and co-workers [72]. The erythrocytes from cow blood were separated by centrifugation and washed with phosphate buffer (0.2 M, pH 7.4) at 800 g for 10 min. The erythrocytes were then diluted with phosphate buffered saline to give 4% suspension. A 500 mg of freeze dried extract in 1 mL of saline buffer was added to a 2 mL of the erythrocyte suspension and the volume was made up to 5 mL with saline buffer. The mixture was incubated for 5 min at room temperature and then 0.5 mL of 0.3% H_2_O_2_ solution in saline buffer was added to induce the oxidative degradation of the membrane lipids. The concentration of H_2_O_2_ in the reaction mixture was adjusted to bring about 90% hemolysis of blood cells after 240 min in the control tubes without the seed extract. After incubation, the reaction mixture was centrifuged at 800 g for 10 min and the extent of hemolysis corresponding to hemoglobin liberation was determined by measuring the absorbance at 540 nm. Inhibitory activity of the different extracts on hemolysis was calculated and expressed as percent inhibition.

#### 3.9.4. Antidiabetic Activity

##### 3.9.4.1. α-Amylase Inhibitory Activity

The α-amylase inhibitory activity of *C. decidua* was determined by a slightly modified method proposed by Bernfeld [73]. Reaction mixture contained 20 μL of α-amylase (0.05 U/μL), 20 μL of sample and 250 μL of 2% starch solution in 0.1 M sodium phosphate buffer (pH 6.9). The reaction was carried out at 37 °C for 10 min and terminated by the addition of 200 μL of DNS reagent (1% 3,5-dinitrosalicylic acid and 12% sodium potassium tartrate in 0.4 M NaOH). The reaction mixture was heated for 15 min at 100 °C and then diluted with 5 mL of distilled water. The α-amylase activity was determined by measuring absorbance at 540 nm by using equation:

$$\text{Inhibition}\%=\left[1-\frac{\left(A_{I}-A_{IB}\right)}{\left(A_{O}-A_{OB}\right)}\right]\times 100$$

where *A_I_* is absorbance of sample solution, *A*_0_ is absorbance of control solution, *A_I_*_B_ is absorbance of blank, and *A*_0B_ is absorbance of control.

##### 3.9.4.2. α-Glucosidase Inhibitory Assay

The enzyme inhibition activity for α-glucosidase was assessed according to the method reported by Matsui and co-workers [74] with minor modifications. 1 mL of 3 mM *p*-nitrophenyl α-d-glucopyranoside (pNPG) in 0.2 M sodium phosphate buffer (pH 6.8) was added as a substrate to the mixture of 50 μL of α-glucosidase (0.15 unit/mL), and 50 μL of sample to start the reaction. The reaction was conducted at 37 °C for 15 min and stopped by the addition of 750 μL of 0.1 M Na_2_CO_3_. The α-glucosidase activity was assessed by measuring the release of *p*-nitrophenol from pNPG at 410 nm. Glucosidase activity inhibition was determined as percentage of control, as follows:

$$\text{Inhibition}\%=100\%-\frac{A_{I}}{A_{0}}$$

where *A_I_* is absorbance of sample solution, and *A*_0_ is absorbance of control solution. Acarbose was used as a positive control.

#### 3.10. Statistical Analysis

All analyses were performed in triplicate and values expressed as the mean ± standard deviation. Data analysis was carried out using the analysis of variance and MSTATC statistical computer package [75].

## 4. Conclusions

The content of biologically active compounds, as well as the potential antidiabetic activity of *Capparis decidua*, has been investigated in this study. The compositional studies indicated *Capparis decidua* seeds as rich sources of all three major food components, *i.e.* carbohydrates, lipids and proteins. Similarly, the amino acid profile indicated sufficient amounts of both essential and non-essential amino acids. Fatty acid analyses indicated that unsaturated fatty acids are present in abundance, while all major tocopherols are present in significant amounts. The tested extracts are rich in phenolic compounds, as well as in glucosinolates, which may contribute to its *in vivo* antidiabetic effect. Furthermore, this study suggests that the glucose lowering effect of this plant can be due, at least in part, to the inhibition of α-amylase. In conclusion, the results from this study contribute to the rational use of *C. decidua* in folk medicine for the treatment of diabetes, by inhibition of α-amylase activity. Further investigations are warranted to identify the active principles and elucidate other possible mechanism(s) of action.

## Figures and Tables

**Table 1:** Proximate chemical composition of seeds of *Capparis decidua.* Data are expressed as the mean ± standard deviation; values having different letters differ significantly (*p* < 0.05).

Component	Percentage ± SD
Crude protein	27.71 ± 1.39 a
Total lipids	29.11 ± 1.07 a
Total carbohydrates	25.42 ± 0.26 a
Crude fiber	10.44 ± 0.09 b
Moisture	4.29 ± 0.14 c
Ash	3.03 ± 0.52 c

**Table 2:** Percentage composition of amino acids in seeds of *Capparis decidua.* Data are expressed as the mean ± standard deviation; values having different letters differ significantly (*p* < 0.05).

Amino acid	Percentage ± SD
Isoleucine	4.03 ± 0.19 c
Leucine	6.41 ± 0.22 c
Lysine	6.02 ± 0.54 c
Methionine	0.75 ± 0.62 d
Phenylalanine	5.51 ± 0.11 c
Threonine	3.64 ± 0.07 c
Tryptophan	0.88 ± 0.05 d
Valine	6.89 ± 0.24 c
Arginine	3.46 ± 0.66 c
Histidine	4.05 ± 0.29 c
Alanine	4.99 ± 0.45 c
Aspartic acid	11.91 ± 0.14 b
Cysteine	0.34 ± 0.01 d
Glutamic acid	24.01 ± 0.56 a
Glycine	4.86 ± 0.39 c
Proline	4.71 ± 0.53 c
Serine	4.40 ± 0.46 c
Tyrosine	2.58 ± 0.95 c

**Table 3:** Fatty acid profile of seeds of *Capparis decidua.* Data are expressed as the mean ± standard deviation; values having different letters differ significantly (*p* < 0.05).

Component	Percentage ± SD
Palmitic acid (16:0)	9.15 ± 1.06 c
Palmitoleic acid (16:1)	4.07 ± 0.23 d
Stearic acid (18:0)	3.89 ± 0.34 d
Oleic acid (18:1)	33.19 ± 0.19 b
Linoleic acid (18:2)	47.33 ± 1.04 a
Linolenic acid (18:3)	1.07 ± 0.35 d
Arachidic acid (20:0)	0.78 ± 0.11 d
Eicosenoic acid (20:1)	0.52 ± 0.38 d

**Table 4:** Tocopherol profile of seeds of *Capparis decidua.* Data are expressed as the mean ± standard deviation; values having different letters differ significantly (*p* < 0.05).

Component	Content (mg/100g) ± SD
α-Tocopherol	9.32 ± 0.41 c
β-Tocopherol	1.04 ± 0.22 d
γ-Tocopherol	744 ± 0.78 a
*δ*-Tocopherol	206 ± 0.83 b
Total	1046.04 ± 0.55

**Table 5:** Percent composition of sterols in seed oil of *Capparis decidua.* Data are expressed as the mean ± standard deviation; values having different letters differ significantly (*p* < 0.05).

Component	Percentage ± SD
Cholesterol	1.02 ± 0.65 e
Brassicasterol	0.19 ± 0.03 e
24-Methylenecholesterol	0.41 ± 0.11 e
Campesterol	21.07 ± 1.02 b
Campestanol	0.36 ± 0.16 e
Stigmasterol	14.30 ± 1.24 c
β-Sitosterol	54.61 ± 1.66 a
Δ^5^-Avenasterol	6.15 ± 0.87 d
Δ^5,24^-Stigmastadienol	0.59 ± 0.23 e
Δ^7^-Stigmastadienol	0.26 ± 0.17 e
Δ^7^-Avenasterol	1.03 ± 0.36 e

**Table 6:** Total glucosinolates (TG) and total phenolic contents (TP) of methanol extract from different organs of *Capparis decidua.* Data are expressed as the mean ± standard deviation; values having different letters differ significantly (*p* < 0.05).

	TG (μmol/g) ± SD	TP (mg CE/g *) ± SD
Stems	107.39 ± 1.57 a	---
Fruits	96.17 ± 2.44 b	407.72 ± 0.81 a
Flowers	84.36 ± 0.98 c	341.12 ± 0.54 b
Roots	77.48 ± 0.59 d	---
Leaves	79.23 ± 1.20 d	286.51 ± 4.62 c

* CE means Catechin Equivalent

**Table 7:** Antioxidant activity of methanol extract from different organs of *Capparis decidua.* Data are expressed as the mean ± standard deviation; values having different letters differ significantly (*p* < 0.05).

	DPPH (IC_50_, μg/mL)	ABTS (μmol TE/g)	FRAP (μmol TE/g)	TRAP (μmol TE/g)	SRSA (IC_50_, μg/mL)
Leaves	104.17 ± 1.68 a	341.86 ± 1.37 c	1132.50 ± 6.64 c	61.86 ± 1.22 a	123.05 ± 0.83 a
Flowers	89.04 ± 1.75 b	466.12 ± 1.08 b	1967.47 ± 4.69 b	55.72 ± 1.93 b	117.20 ± 1.29 b
Fruits	69.1 ± 1.3 c	501.17 ± 1.34 a	2388.02 ± 1.48 a	47.04 ± 2.08 c	93.04 ± 1.63 c

**Table 8:** α-Amylase, α-glucosidase and antihemolytic activity of methanol extracts from *Capparis decidua.* Values having different letters differ significantly (*p* < 0.05); Nd = not determined.

	Inhibition ratio (%)		
Methanol extract	α-amylase	α-glucosidase	antihemolytic activity
Leaves (100 μg/mL)	46 a	39 b	47 c
Flowers (100 μg/mL)	50 c	47 a	53 b
Fruits (100 μg/mL)	61 b	56 c	61 a
Leaves (500 μg/mL)	75 c	78 a	Nd
Flowers (500 μg/mL)	84 b	85 c	Nd
Fruits (500 μg/mL)	96 a	91 b	Nd
